# Effect of immobilized fungal phytase on growth performance and bone traits of broilers fed with low dietary calcium and phosphorus

**DOI:** 10.14202/vetworld.2018.758-764

**Published:** 2018-06-07

**Authors:** Sreeja Ajith, Divya Shet, Jyotirmoy Ghosh, Vaibhav B. Awachat, Karthik Bhat, Dintaran Pal, Arumbackam V. Elangovan

**Affiliations:** 1 ICAR-National Institute of Animal Nutrition and Physiology, Bengaluru, Karnataka, India; 2 Department of Microbiology, Jain University Bengaluru, Karnataka, India; 3 Department of Biotechnology, Jain University, Bengaluru, Karnataka, India

**Keywords:** broiler, calcium, phosphorus, phytase

## Abstract

**Aim:**

The aim of this study was to investigate the effects of phytase which was laboratory produced by *Aspergillus foetidus* on the growth performance, mineral retention, and bone traits of broilers fed with low dietary calcium and phosphorus.

**Materials and Methods:**

The extracellular phytase enzyme secreted into the crude filtrate was concentrated by ammonium sulfate precipitation to obtain an activity of 500 phytase units (FTU). A total of 90 1-day-old chicks (Cobb 500) were randomly divided into three treatment groups with five replicates having six birds each. Dietary treatment, T1, was with 0.45% non-phytate P (NPP) during starter and 0.40% during finisher phase with 1% Ca. Dietary treatment, T2, had 0.37% NPP during starter and 0.32% in finisher phase with 1% Ca and supplemental lab phytase at 500 FTU/kg. Dietary treatment, T3, was similar to T2 with a lower Ca of 0.8%.

**Results:**

There was no significant difference among the dietary treatments with regard to body weight gain, feed intake, feed conversion ratio, and Ca retention (p>0.05). However, a significant improvement in retention of P by birds was observed in phytase supplemental groups T2 and T3 (p<0.05). Dry weight of tibia (2.58-2.78 g/kg live weight) and ash content (39.7-41.8%) was comparable among treatments. A similar trend was observed for bone Ca, P, and Mn content.

**Conclusion:**

The study indicated that 500 FTU/kg phytase can be effectively supplemented in a broiler diet with low phosphorus (0.37% in starter and 0.32% NPP in finisher diet) and low calcium (0.8% in diet) for better growth performance and with successful replacement of dietary P by 0.08 % and reduced P excretion into the environment in broiler chicken.

## Introduction

Phytase is a phosphomonoesterase capable of hydrolyzing phytate (inositol hexaphosphate IP6) to inorganic phosphate and other minerals that are readily absorbed by the monogastric animals [1]. Phytase is globally accepted as feed enzyme for monogastric animals and has been a crucial area for poultry and pig research. The upshot of phytase over P availability and its advantages are well established which enlightens on improved weight gain, mineral retention, metabolizable energy, and amino acid digestibility in poultry [2-5]. Positive effects of microbial phytase were reported on the digestibility of Ca, Mg, Na, and K [6,7]. The bioavailability of P and other minerals such as Ca, Cu, and Zn due to the addition of phytase is also well recognized [8-10]. Literature studies over the past two decades have substantiated the advantages of phytase and have rapidly become a nutritional strategy to compact with the environmental and economic pressure.

Although the benefits of phytase to improve P and Ca digestibility are universally accepted and have been validated with several publications, their ratios in the feed formulation are not exploited much. Broiler diets are typically formulated for 10 g of Ca/kg diet [11]. Ca has a lower affinity with phytate unlike other minerals; however, its higher concentration (higher than 1% in diet) plays the decisive role in determining the phytate-mineral complex [12]. The studies of Tamim and Angel [13] have reported on the negative effects of high Ca in phytase-mediated phytate hydrolysis in broilers. Increased dietary Ca hinders the action of phytase by forming insoluble Ca-phytate complexes in the digestive tract [14]. It also results in Ca competing for the active sites of phytase leading to a direct reduction of phytase activity. Reducing the ratio of dietary Ca to available P positively affects the P digestibility [15]. Contrary to the above finding, increasing the Ca level from 6.7 to 13.3 g/kg did not affect phytase activity [16]. Delezie *et al*. [17] observed that reducing Ca and P concentrations by 20% with phytase supplementation could enhance broiler performance. Rama Rao *et al*. [18] stated that the Ca and P coexist in many biological functions though the dietary requirements are interdependent. Surplus calcium in the diet has been shown to increase the pH of the gizzard, digesta, and reduced phosphorous absorption by forming insoluble complexes in the intestinal lumen [19]. Lower calcium levels travail bone mineralization, causing excess phosphorous wastage through droppings. Hence, Ca:P ratio in the diet is essential for the better utilization of both Ca and P.

Several studies have confirmed the deleterious effect of excess Ca on phytase activity; however, there is a need to explore and define appropriate dietary non-phytate P (NPP) and Ca levels in poultry diet supplemented with phytase. Thus, the present study was conducted to understand the growth performance, mineral retention, and bone traits of broiler chicken at low P and Ca supplemented with laboratory produced fungal phytase by *Aspergillus foetidus* by immobilization technique. *A. feotidus* was employed for continuous, economic phytase production with improved stability and longer shelf life.

## Materials and Methods

### Ethical approval

All the experimental procedures and animal trial were executed with the approval of Ethical Committee of ICAR-National Institute of Animal Nutrition and Physiology, Bengaluru (Animal care and use protocol (No. 5/2012)).

### Phytase production

*A. foetidus* MTCC 11682 was used for phytase production employing immobilization technique of adsorption. A thoroughly homogenized spore suspension of 5% w/v inoculum, extracted in 0.1% tween 20, and physiological saline in the ratio of 1:5 were immobilized on polyurethane foam matrix. A scale-up phytase production was executed in an optimized media following the techniques of Lalpanmawia *et al*. [20]. The crude phytase obtained in repeated batch fermentation was filtered, centrifuged (10,000 rpm for 15 min), and precipitated using 90% ammonium sulfate. The precipitates were liquefied in 0.2M sodium citrate buffer, pH 5.5. They were further concentrated and aliquoted to screw cap tubes in such a way that each tube had an activity of 500 FTU. The aliquots were stored at −20°C for feeding trails. After thawing, the concentrated phytase of 500 FTU was added to 1 kg feed and mixed well.

### Birds and housing

A 90 one-day-old, unsexed (maybe 50:50), Cobb 500 chicks were distributed into three treatment groups with five replicates under each treatment with six birds in each replicate in a completely randomized design (30 birds per treatment). The chicks were housed in battery cages (72 cm wide × 48 cm long × 38 cm high) as groups in a random manner allotted tiers from 0 to 4 weeks of age. Each battery cage was equipped with heating arrangements, feeders, drinkers, and dropping trays. The cages were placed in a well-ventilated open-sided house with 24 h lighting. Temperature inside the cage was maintained at 33°C at the start and was gradually reduced to 25°C. The room temperature was around 24-25°C with a relative humidity of 50-60%. Feed and fresh water was provided *ad libitum*. The experiment was conducted for 28 days.

### Experimental design

Treatment 1 (T1) consisted of standard available phosphorus (AP) with 0.45% NPP during starter (0-21 days) and 0.40% during the finisher (21-28 days) phase along with 1% Ca throughout the growing phase. Treatment 2 (T2) was with low phosphorus (0.37% during starter and 0.32% in finisher phase) with phytase at 500 FTU/kg diet and 1% Ca. Treatment 3 (T3) was a low phosphorus (0.37% during starter and 0.32% in finisher phase) and low calcium of 0.8% with phytase at 500 FTU/kg diet. The details of the ingredient and nutrient composition are briefed in Table-1.

**Table-1:** Ingredient and nutrient composition of experimental diets during starter (0-3 weeks) phase and finisher phase (3-4 weeks).

Ingredients	Starter (0-3 weeks)			Finisher (3-4 weeks)		
	
	T1	T2	T3	T1	T2	T3
Ingredient composition as fed basis (%)						
Maize	56.97	57.12	57.62	63.40	63.60	64.15
Soybean meal	37	37	37	31	31	31
Sunflower oil	2.2	2.2	2.2	2	2	2
Lime stone	1	1.3	0.8	1.2	1.5	0.95
DCP	1.75	1.3	1.3	1.5	1	1
Salt	0.35	0.35	0.35	0.35	0.35	0.35
Lysine	0.3	0.3	0.3	0.2	0.2	0.2
Methionine	0.18	0.18	0.18	0.1	0.1	0.1
Vitamin premix*	0.25	0.25	0.25	0.25	0.25	0.25
Phytase (FTU)	0.0	500	500	0.0	500	500
Nutrient composition (g/kg)						
ME, kcal/kg	2996	2996	3012	3050	3055	3071
CP	22.3	22.3	22.4	20.09	20.10	20.15
Lysine	1.44	1.44	1.44	1.15	1.15	1.15
Methionine	0.49	0.49	0.49	0.46	0.46	0.46
Ca	1.00	1.01	0.82	1.01	1.01	0.80
P, avail.	0.45	0.37	0.37	0.40	0.32	0.32

* Trace mineral premix, 1 g/kg; Vitamin premix, 1 g/kg; and Choline 0.5g/kg. Trace mineral premix supplied mg/kg diet: Mg, 300; Mn, 55; I, 0.4; Fe, 56; Zn, 30; and Cu, 4. The vitamin premix supplied per kg diet: Vitamin A, 8250 IU; Vitamin D3, 1200 ICU; Vitamin K, 1 mg; Vitamin E, 40 IU; Vitamin B1, 2 mg; Vitamin B2, 4 mg; Vitamin B12, 10 mg; niacin, 60 mg; pantothenic acid, 10 mg; and choline, 500 mg

### Growth performance

Body weight (BW) and feed intake (FI) were recorded on weekly basis to calculate feed conversion ratio (FCR) on the basis of unit feed consumed to unit BW gain (BWG).

### Metabolic trial

A 3-day metabolic trial was conducted on day 23. Measured amount of feed was offered, and daily feed consumption and residual feed were recorded on cage basis. Excreta voided were collected at the end of metabolic trial from each replicate, homogenized thoroughly, and weighed. Representative samples of the excreta were dried in an oven at 60°C, grounded uniformly, and ashed in muffle furnace at 600°C. The ashed samples were digested with dilute hydrochloric acid (HCl), and the extract was used for mineral estimation using inductively coupled plasma optical emission spectroscopy (ICP-OES), a PerkinElmer instrument, Waltham, Massachusetts, US. All calculations were expressed on dry matter (DM) basis. The difference in the nutrient content of the feed consumed and of the droppings was used to calculate the retention of nutrients (Ca and P) on percentage DM basis.

### Carcass quality

At the end of the experiment, 10 birds per treatment (2 birds per group) were sacrificed randomly by cervical dislocation. The carcass weight and weight of liver, gizzard, and heart were recorded.

### Bone morphometry and mineral estimation

The left tibia was collected from the sacrificed birds. The adhering tissues were cleaned and dried. Total length and proximal and distal epiphysis and diaphysis width were measured using a digital Vernier caliper. Tibia was defatted in petroleum ether (boiling point 60-80°C) using Soxhlet apparatus, dried to a constant weight in a drying oven, and ashed for 12 h at 600°C. The ashed samples were digested with dilute HCl, and the mineral extract prepared was used for the estimation of Ca, P, Mn, and zinc (Zn) using ICP-OES.

### Phytate and phytase estimation

Phytate P content of feed was estimated as described by Haugh and Lantzsch [21]. Phytase estimation was as per Kim and Lei [22].

### Statistical analysis

The experimental data were subjected to one-way analysis of variance for fully randomized design and tested for significance among the three treatments using Turkey’s ASD *post hoc* test (SPSS-2010 version 18.0).

## Results

### Growth performance

BWG, FI, and FCR did not differ significantly (p>0.05) among the three treatment groups during the entire phase (Table-2). Carcass traits (eviscerated and giblet weight) were not influenced (p>0.05) by dietary AP and/or phytase supplementation (Table-3).

**Table-2:** Growth performance of broiler chicken.

Groups	BWG (g/b)		FI (g/b)		FCR	
		
	0-3 weeks	0-4 weeks	0-3 weeks	0-4 weeks	0-3 weeks	0-4 weeks
T1	814	1346	1129	1843	1.32	1.37
T2	841	1349	1134	1876	1.28	1.39
T3	809	1293	1111	1834	1.31	1.42
SEM	13.42	14.28	7.68	13.82	0.023	0.016
Significance	0.62	0.21	0.45	0.45	0.82	0.53

SEM=Standard error of mean, T1=Control group, T2=Low *P+*500 FTU lab phytase, T3=Low P, low Ca+500 FTU lab phytase. BWG=Body weight gain, FI=Feed intake, FCR=Feed conversion ratio

**Table-3:** Influence of dietary treatment on the carcass traits of broilers.

Carcass Traits (% of live weight)	T1	T2	T3	SEM	Significance
Eviscerated	60.06	60.17	59.99	0.34	0.98
Liver	2.30	2.33	2.21	0.04	0.43
Gizzard	2.24	2.19	2.38	0.06	0.43
Heart	0.62	0.67	0.64	0.013	0.21
Giblet	5.16	5.19	5.23	0.073	0.94

SEM=standard error of mean, T1=Control group, T2=Low *P+*500 FTU lab phytase, T3=Low P, low Ca+500 FTU lab phytase

### Mineral and DM retention

Phytase supplementation did not show any significant improvement (p>0.05) in DM and calcium retention (Table-4). A significantly (p<0.05) higher retention of P by birds was observed in T2 (low AP level with phytase supplementation at 500 FTU/kg) and T3 (lower P and Ca with phytase at 500 FTU/kg).

**Table-4:** Influence of dietary treatments on the retention (%) of dietary minerals.

Retention (%)	Groups			SEM	Significance

	T1	T2	T3		
DM	62.6	62.0	59.4	0.012	0.46
Ca	36.5	39.8	39.9	0.017	0.68
P	42.2^b^	49.3^a^	51.1^a^	0.015	0.02

a,b Means with different superscripts in a row differ significantly (p<0.05). SEM=Standard error of mean, T1=Control group, T2=Low *P+*500 FTU lab phytase, T3=Low P, low Ca+500 FTU lab phytase. DM=Dry matter

### Bone morphometry and mineralization

The bone weight, bone length, and bone width were similar (p>0.05) among the treatment groups (Table-5). Dry weight of tibia did not show any significant difference (p>0.05) among treatments. The tibia bone had similar (p>0.05) ash contents among the three dietary treatments of birds, and the same trend was also observed for Ca, P, and Mn content of the ash (Table-6). Supplementation of phytase at 500 FTU/kg did not increase the Zn content in T2 and T3. On the contrary, the control treatment showed an increased concentration of Zn in bone ash (p<0.05).

**Table-5:** Bone morphometry (left tibia) of broiler chicken on different dietary treatment.

Treatment	Bone weight (g/kg live weight)	Bone length (mm/kg live weight)	Bone width (mm/kg live weight)		

			Proximal epiphysis	Diaphysis	Distal epiphysis
T1	2.78	55.73	13.14	5.12	10.36
T2	2.69	54.59	13.56	5.16	11.13
T3	2.58	54.12	13.29	5.42	10.61
SEM	0.04	0.82	0.17	0.09	0.19
Significance	0.17	0.73	0.62	0.35	0.27

SEM=Standard error of mean, T1=Control group, T2=Low *P+*500 FTU lab phytase and T3=Low P, low Ca+500 FTU lab phytase

**Table-6:** The effect of dietary treatments on bone mineralization.

Treatment	Ash (%)	Calcium (%)	Phosphorus (%)	Zinc (ppm)	Manganese (ppm)
T1	41.8	19.45	10.39	229.4^b^	138.7
T2	40.7	18.04	9.88	162.2^a^	136.4
T3	39.7	18.72	10.17	165.0^a^	141.8
SEM	0.01	0.47	0.25	11.34	3.16
Significance	0.36	0.49	0.72	0.02	0.79

a,b Means with different superscripts in a row differ significantly (p<0.05). SEM=Standard error of mean, T1=Control group, T2=Low *P+*500 FTU lab phytase, T3=Low P, low Ca+500 FTU lab phytase

## Discussion

Phytase supplementation in low-P diet (0.30-0.38 AP in diet) increases growth performance in broiler chicken [23]. Limited studies are available on broiler trials performed with laboratory produced phytase [20,24-26]. Ahmad *et al*. [24] observed comparable results between phytases produced from *Aspergillus niger*, supplemented at 500 FTU/kg diet and normal P diet group in his 28-day trial. They reported a BWG of 869 versus 856 g/bird, FI of 1187 versus 1137 g/bird, and FCR of 1.36 versus 1.33. In the comparative study of Lan *et al*. [25] between birds fed on low AP diet supplemented with freeze-dried active *Mitsuokella jalaludini* culture, a rumen bacteria and natuphos phytase both equivalent to 500 FTU/kg diet observed similar BWG (784 vs. 797 g/bird), lower FI (1015 vs. 1058), and significantly better FCR (1.38 vs. 1.41). However, the studies of Lalpanmawia *et al*. [20] reported a lower growth rate (1598 vs. 1744 g/bird), a lower FI (2576 vs. 2808), and a similar FCR (1.61 vs. 1.63) in broiler fed with low AP complementing with 500 FTU/kg laboratory produced phytase compared to normal P-fed group. Although the findings of Manobhavan *et al*. [26] highlight on the benefits of phytase superdosing up to 5000 FTU/kg over the standard 500 FTU/kg diet, similar BWG (1642 vs. 1669), FI (2634 vs. 2697), and FCR (1.61 vs. 1.62) were reported for broilers fed on low AP diet with lab phytase supplemented at 500 FTU/kg in comparison with chickens fed on normal P. The current study is in agreement with the results of the previous findings, exhibiting in par performance of normal P to low P group supplemented with phytase at 500 FTU/kg. The comparable growth performance observed in all the three dietary treatments might be due to the release and utilization of P from the phytate mineral complex [27]. A better FCR (1.96 vs. 2.08) was observed with phytase supplementation at 500 FTU when compared to non-supplemented group [28]. Similarly, Akter *et al*. [29] reported on a better FCR in birds supplemented with phytase and medium Ca diet. However, Rutherfurd *et al*. [30] studied that there was no effect on the FI:BWG for the birds fed on low P diet supplemented phytase at 1000 and 2000 FTU/kg compared with non-supplemented low P diet, and performance was equal to that of the normal AP diet. Supplemental phytase aids in releasing bound organic nutrients and removing the negative effects of phytic acid on proteolytic enzymes leading to increased DM digestibility [7]. The higher P retention observed in the phytase supplemented group can be credited to the increased P bioavailability [31] and the homeostasis mechanism exhibited by broiler that increases retention and absorption of P at low dietary intake when compared to normal dietary P. Sebastian *et al*. [27] observed an increase in Ca and P retention in chicken by 12.4 and 12.2%, respectively, when fed with corn soya-based diet (low P level 0.5%) supplemented with phytase at 600 FTU/kg.

Chung *et al*. [32] reported that phytase supplementation had no significant impact on tibial ash from broiler, which is in complete agreement with the current study. Various studies have substantiated the improved bone mineral content, bone density, and bone breaking strength with phytase supplementation [27,33]. Phytase resulted in better mineralization of bone by improving the availability of Ca and P. Kiarie *et al*. [34] reported on the role of phytase in improved digestibility of minerals such as Ca and Mg other than P, due to decreased phytate-Ca complex formation in the gastrointestinal tract of poultry. The comparable results for Ca-P retention obtained in phytase supplemental group to that of standard AP indicate phytate P utilization by microbial phytase and the release of Ca from Ca-phytate complex. Phytase along with inorganic P intensifies bone length, tibia ash, tibial strength, and mineralization of cartilage and bone cells [35]. In an experiment with 30-day-old broilers fed on corn-soybean diet, a reduction in tibiotarsus ash content was found when the Ca and NPP levels of the diets decreased from 10-4.5 to 7.5-3 g/kg and the content was not affected by phytase supplementation [36]. The beneficial effects of phytase might be attributed to the release of minerals from complexes with phytic acid and promoting the deposition of minerals in bone [23]. The increase in Zn concentration in bone ash exhibited by control group could be due to some antagonistic effect; however, the exact reason is not known. 

There is a need to demarcate the proportionate Ca level and Ca: P ratio in phytase supplemented diets for broilers [12]. The studies of Qian *et al*. [37] recommend Ca: P ratio in the range of 1.1-1.4: 1 for broilers. The report also says that higher levels of Ca (5.61-10.20 g/kg) and Ca: P ratio of 1.1-2.0:1 had a negative impact on the weight gain (420 g/bird vs. 553 g/bird) of 21-day-old broiler. However, phytase supplementation at 900 FTU/kg boosted the weight gain in broilers fed on higher (541 g/bird vs. 420 g/bird) and lower (615 g/bird vs. 553 g/bird) Ca: P ratio [37]. Applegate *et al*. [38] reported that conventionally used dietary Ca levels in broiler diets (0.9%) resulted in the reduction of intestinal phytase activity and in apparent ileal PP hydrolysis compared with a lower level of Ca (0.4%). Increasing Ca concentration in phytase-supplemented diets caused a linear decrease in the BWG, FI, and feed efficiency in broiler chicken [39]. Tamim *et al*. [40] reported that limited dietary Ca could initiate endogenous phytases to hydrolyze 80% phytate in excess. This was substantiated by the studies of Wilkinson *et al*. [41], who reported on the synchronized assimilation of Ca-phytate and phytate hydrolysis by endogenous phytase attained by spatial separation of Ca from the grain portion of the diet. Li *et al*. [42] supported the proposal that low P levels upregulate mucosal phytase activity which should be facilitated by low Ca levels. Zeller *et al*. [43] reported on the level of intestinal IP6 desertion and iP addition. They observed a hydrolysis of IP6 by 67% in the jejunum and 78% in the ileum in a corn-soybean diet without monocalcium phosphate and supplemented *Escherichia coli* phytase at 500 FTU/kg diet. Dietary Ca level between 5 and 7 g/kg would be insufficient for bone mineralization irrespective of phytase supplementation [44]. The present study observed lowering of Ca level up to 0.8% in starter and finisher diets with low NPP and supplemented phytase at 500 FTU/kg diet. However, dietary Ca reduction should be proportional to total P reduction maintaining Ca:P ratio as observed by Qian *et al*. [37].

## Conclusion

The study indicated that 500 FTU/kg phytase can be effectively supplemented in a broiler diet with low phosphorus (0.37% in starter and 0.32% NPP in finisher diet) and low calcium (0.8% in diet) successfully replacing 0.08% of dietary P and reduced P excretion into the environment.

## Authors’ Contributions

AVE designed and supervised the experiment. SA executed the experiments and wrote the manuscript. DS and VBA helped in conducting the experiments. KB and DP helped in the ICP work. JG and AVE corrected the manuscript. All authors have read and approved the manuscript.
